# Marital status and risk of dementia over 18 years: Surprising findings from the National Alzheimer's Coordinating Center

**DOI:** 10.1002/alz.70072

**Published:** 2025-03-20

**Authors:** Selin Karakose, Martina Luchetti, Yannick Stephan, Angelina R. Sutin, Antonio Terracciano

**Affiliations:** ^1^ Florida State University College of Medicine Tallahassee Florida USA; ^2^ University of Montpellier Euromov UFRSTAPS Montpellier France

**Keywords:** Alzheimer's disease, cognitive impairment, dementia, marital status

## Abstract

**INTRODUCTION:**

Marital status is a potential risk/protective factor for adverse health outcomes. This study tested whether marital status was associated with dementia risk in older adults.

**METHODS:**

Participants (*N* = 24,107; Mean_age _= 71.79) were from the National Alzheimer's Coordinating Center. Cox regressions tested the association between baseline marital status and clinically ascertained dementia over up to 18 years of follow‐up.

**RESULTS:**

Compared to married participants, widowed (hazard ratio [HR] = 0.73, 95% confidence interval [95% CI] = 0.67–0.79), divorced (HR = 0.66, 95% CI = 0.59–0.73), and never‐married participants (HR = 0.60, 95% CI = 0.52–0.71) were at lower dementia risk, including for Alzheimer's disease and Lewy body dementia. The associations for divorced and never married remained significant accounting for demographic, behavioral, clinical, genetic, referral source, participation, and diagnostic factors. The associations were slightly stronger among professional referrals, males, and relatively younger participants.

**DISCUSSION:**

Unmarried individuals may have a lower risk of dementia compared to married adults. The findings could indicate delayed diagnoses among unmarried individuals or challenge the assumption that marriage protects against dementia.

**Highlights:**

- Widowed, divorced, and never‐married older adults had a lower dementia risk, compared to their married counterparts.
- Unmarried older adults were also at a lower risk of Alzheimer's disease and Lewy body dementia, with a pattern of mixed findings for frontotemporal lobar degeneration, and no associations with risk of vascular dementia or mild cognitive impairment.
- All unmarried groups were at a lower risk of progression from mild cognitive impairment to dementia.
- There was some evidence of moderation by age, sex, and referral source. However, stratified analyses showed small differences between groups, and most interactions were not significant, suggesting that the role of marital status in dementia tends to be similar across individuals at different levels of dementia risk due to education, depression, and genetic vulnerability.

## 1 BACKGROUND

Being married has been related to better health and longevity.^1^, ^2^ These associations are often explained through the marital resource model, which suggests that marriage provides social, psychological, and economic benefits unavailable through other relationships that, in turn, support healthier and longer lives.^3^, ^4^ This association may extend to risk for cognitive impairment and dementia, yet there is significant heterogeneity in cognitive health across marital status.^5^, ^6^, ^7^, ^8^, ^9^, ^10^

There is some evidence of a higher risk of Alzheimer's disease (AD) and related dementias among never‐married individuals, compared to married ones.^5^, ^11^ Null results have also been reported.^10^ The evidence is also mixed for divorce and widowhood: While two studies reported that both divorced and widowed individuals have a higher dementia risk compared to their married counterparts,^5^, ^6^ a meta‐analysis found that widowed individuals, not divorced ones, had a significantly higher risk of dementia compared to married individuals.^8^ Divorce, in some cases, can lead to increased happiness^12^ and life satisfaction,^13^ which may potentially protect against dementia risk.^14^, ^15^ Indeed, a recent study reported that divorce was associated with a slower rate of cognitive decline.^16^ Mixed results have also occurred for specific causes of dementia: One study found that divorced and widowed categories were associated with a greater risk of AD,^17^ but other studies have found no associations with AD or vascular dementia (VD).^8^, ^11^ The association between marital status and Lewy body dementia (LBD) and frontotemporal lobar degeneration (FTLD) is untested. The evolving role of marriage in society and the rising number of unmarried (divorced, widowed, or never‐married) individuals,^18^ make it critical to examine whether unmarried older adults are vulnerable to developing dementia. Such research may help identify subgroups of individuals at higher risk who may need closer monitoring and support.

Unmarried males are at a higher risk for poor health outcomes than unmarried females.^19^, ^20^ However, sex differences in the association between marital status and incident dementia is contradictory: One study has reported that both divorce and widowhood increased the risk of dementia more among males compared to females,^5^ and others have reported an association with either widowhood^21^ or divorce,^6^, ^22^ or found no such effect.^23^ Compared to sex, less is known about whether depression and genetic vulnerability moderate the association between marital status and dementia. The risk of dementia among unmarried adults could be pronounced in individuals with higher depressive symptoms or a genetic predisposition to dementia.

Married individuals are more likely to engage in preventive medical care than unmarried individuals,^24^ potentially due to partners who notice early symptoms. Individuals in the early stages of dementia may not be aware of their symptoms and may miss/delay the diagnosis,^25^ especially among unmarried individuals who may lack feedback from a close partner. Indeed, a meta‐analysis suggests that registry‐based studies (e.g., hospital and death records) underestimate dementia risk among widowed and never‐married individuals.^8^ Based on the importance of referral source and seeking medical help (i.e., referred by a professional and clinical evaluation) on cognitive outcomes,^26^, ^27^ it is critical to evaluate whether the association between marital status and risk of dementia differs depending on the referral sources (i.e., self/relative/friend vs. referred by professionals) and the reason for visiting the center (i.e., clinical evaluation vs. research participation).

This study extended prior work in fundamental ways: First, the study used data from the National Alzheimer's Coordinating Center (NACC), one of the largest cohorts with annual clinical evaluation of dementia over one of the longest follow‐ups (up to 18 years). The comprehensive clinical assessment and long follow‐up increase power and reduce the risk of reverse causality (e.g., individuals in the preclinical or prodromal stage of dementia are more likely to get divorced).^23^ Second, the study investigates whether the association differs across cause‐specific dementia (AD, LBD, VD, FTLD), and whether it extends to mild cognitive impairment (MCI). Third, the study investigates whether the associations were moderated by sex, age, race, education, depression, diagnostic form, participant's referral source, primary reason for visiting the Alzheimer's Disease Research Centers (ADRCs), and genetic vulnerability to identify contexts or subgroups with higher vulnerability.

## 2 METHODS

### 2.1 Participants and procedure

The NACC is an ongoing longitudinal study that has enrolled > 50,000 participants, with some up to 19 annual visits. Referral‐based or volunteer participants were recruited from > 42 ADRC across the United States. Since 2005, the Uniform Data Set (UDS) has been collected annually (within a ± 6‐month window) using a standardized protocol for all participants by trained clinicians or clinic staff. Data are gathered through direct observation and/or examination of participants and their co‐participants and is recorded directly on UDS forms during the assessment (see more at: https://naccdata.org/requesting‐data/nacc‐data). All participants and co‐participants provide informed consent before each assessment, and each contributing ADRC maintains their own separate institutional review board approvals from their institutions before submitting data to NACC.

**FIGURE 1 alz70072-fig-0001:**
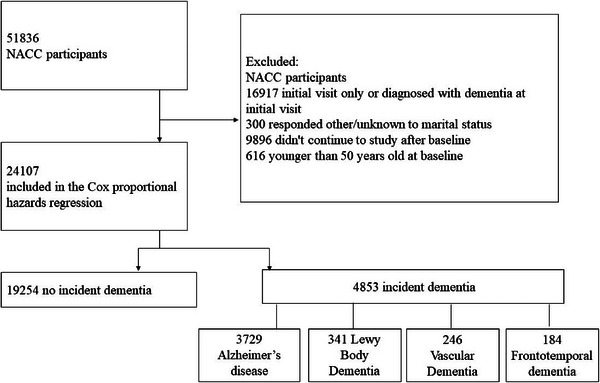
Flowchart of study participant selection. NACC, National Alzheimer's Coordinating Center.

The analyses used the NACC data freeze of June 2024. Exclusion criteria were individuals who withdrew from the study after baseline (*n* = 9896), were younger than 50 (*n* = 616), had dementia diagnosis at baseline (*n* = 16,917), and responded “other/unknown” to marital status at baseline (*n* = 300). The final sample included 24,107 individuals (59.6% female, mean age = 71.79 years) with data on marital status and without dementia at baseline. The flowchart of the selection of study participants is shown in Figure 1.

### 2.2 Measures

#### 2.2.1 Marital status

Marital status at baseline was reported as married, widowed, divorced, separated, never married (or marriage was annulled), or living as married/domestic partners. Because of the small numbers, we combined the separated (*n* = 261) with the divorced group (hereafter “divorced”) and the living as married/domestic partner (*n* = 423) with the married group (hereafter “married”). This married group was the reference group for the main analyses.

**RESEARCH IN CONTEXT**

1. **Systematic review**: Being married has been related to better health and longevity. This association may extend to risk of cognitive impairment and dementia, yet there is significant heterogeneity in cognitive health across marital status.
2. **Interpretation**: This 18‐year cohort study of older adults, based on the National Alzheimer's Coordinating Center Uniform Data Set, found that, compared to married participants, widowed, divorced, and never‐married older adults had a lower dementia risk, including Alzheimer's disease and Lewy body dementia. The findings could indicate delayed diagnoses among unmarried individuals or challenge the assumption that marriage protects against dementia.
3. **Future directions**: Future research is needed to (a) identify the specific pathways that lead to reduced dementia risk among unmarried older adults over time in a more representative sample, and (b) examine the association by considering the duration of post‐marital life and the role of relationship factors (e.g., marital quality).

#### 2.2.2 Incident cognitive impairment and dementia

At each annual visit, trained clinicians assessed cognitive status using neuropsychological tests and clinical examinations. Diagnoses[Fig alz70072-fig-0001] of MCI and dementia were determined by either clinicians or a consensus panel who evaluated whether participants met the criteria for MCI or dementia (0 = no, 1 = yes). Participants with cognitive impairment who did not clearly meet the criteria for MCI or dementia were classified as cognitively impaired–not MCI. Clinicians were also asked to mark the primary etiological diagnoses for participants diagnosed with dementia, including AD, LBD, VD, and FTLD.

The dementia criteria and neuropsychological test battery changed over UDS versions. The dementia diagnosis in the currently used version, UDS Form 3 (implemented in March 2015), was based on the Clinical Dementia Rating (CDR).^28^ CDR training was required for ADRC personnel and provided by the Washington University ADRC (https://knightadrc.wustl.edu/cdr‐training‐application/). In earlier versions 1.2 (September 2005–February 2008) and 2 (until March 2015), specific diagnostic criteria for dementia were not provided. However, most centers used the Diagnostic and Statistical Manual of Mental Disorders–Fourth Edition^29^ criteria for dementia (See Researchers Data Dictionary: https://naccdata.org/). For the neuropsychological test battery, with UDS Form 3, the Mini‐Mental State Examination (MMSE) was replaced by the Montreal Cognitive Assessment (MoCA), Wechsler Memory Scale Revised (WMS‐R) Logical Memory was replaced by Craft Story Recall, WMS‐R Digit Span was replaced by Number Span, and the Boston Naming Test was replaced by the Multilingual Naming Test (MINT).

#### 2.2.3 Covariates and moderators

Demographic covariates included the participant sex (0 = female, 1 = male), baseline age (in years), race (three variables: Asian = 1 vs. other = 0; Black = 1 vs. other = 0; White = 1 vs other = 0), Hispanic/Latino ethnicity (0 = no, 1 = yes), education (in years), and living alone (0 = not alone, 1 = live alone). Follow‐up analyses included physical (obesity, diabetes, hypertension), mental (depression) health, behavioral (smoking), and genetic vulnerability assessed at baseline because these factors have been used in previous studies^5^, ^14^, ^23^, ^30^ and are associated with marital status and/or dementia.^1^, ^31^, ^32^, ^33^, ^34^, ^35^ Body mass index (BMI) was calculated from staff‐assessed body weight divided by height squared and was used to classify obesity (BMI ≥ 30; 0 = no, 1 = yes). Participants reported diagnoses of diabetes and hypertension (0 = no, 1 = yes). Depression was assessed with the item “Active depression in the last two years” (0 = no, 1 = yes). Current and former smoking status was measured with the items “Average number of packs smoked per day” (0 = no reported cigarette use, 1 = reported cigarette use) and “If the subject quit smoking, age at which he/she last smoked” (0 = not applicable, no significant smoking history, 1 = smoking history). Genetic vulnerability was based on apolipoprotein E (*APOE*) ε4 carrier status (0 = no ε4 allele, 1 = ε4 allele present). We included the diagnostic form (0 = UDS Form 1 and Form 2, 1 = Form 3) to examine whether the associations were dependent on the changes in diagnostic criteria, such as the use of the CDR in the UDS Form 3. Referral source (0 = non‐professional contact: self/relative/friend, 1 = professional contact: clinician, nurse, doctor, other health‐care provider, or other staff [ADRC and non‐ADRC]), and participant's primary reason for coming to ADRC (0 = to participate in a research study, 1 = to have a clinical evaluation/both to participate in a research study and to have a clinical evaluation, or another reason) were included as additional covariates and moderators as enrollment factors may differ across marital status (e.g., married individuals could be more likely to be referred by a professional due to the potential “spousal effect” in seeking health care).^36^

### 2.3 Statistical approach

Cox proportional hazards regression was used to determine whether marital status was associated with the risk of incident dementia. Data from September 2005 to June 2024 were used in the analyses. Survival time was calculated in years from the baseline assessment to the date of earliest dementia diagnosis. Participants who did not develop dementia were censored at their last visit date. We examined the risk of dementia for “unmarried” groups (widowed, divorced, never‐married) compared to the married group (reference). We tested Model 1 with age and sex as covariates. A fully adjusted Model 2 added race, ethnicity, education, living alone, physical health (diabetes, hypertension, obesity), depression, smoking behavior (current smoking and former smoking), diagnostic form, referral source, primary reason to visit ADRC, and genetic vulnerability. We repeated the primary analyses to evaluate the associations with each specific type of dementia (AD, LBD, VD, FTLD). We next examined whether demographics (sex, age, race, education), depression, diagnostic form, enrollment factors (referral source and primary reason to visit ADRC), and genetic vulnerability moderated the association between marital status (married vs. unmarried) and dementia by testing interaction terms in the basic model that also included the main effects, age, and sex. We then ran sensitivity analyses to examine the robustness of the findings. First, considering the median follow‐up time (3.97 years), participants were stratified into those with follow‐ups < 4 years from baseline (Model 1.1) and ≥ 4 years (Model 1.2). Second, a competing‐risks survival regression using a Fine and Gray^37^ semi‐parametric proportional hazards model tested the robustness of the association accounting for age, sex, and the competing risk of death. Third, we excluded participants with MCI and cognitive impairment–not MCI at baseline. Fourth, we examined marital status as a predictor of progression from any MCI to dementia. Fifth, we examined the associations between marital status and incident MCI. Last, based on the evidence linking marital transitions and cognitive health,^16^, ^38^ we first excluded participants who experienced marital transitions over the study follow‐up (*N* = 2916) and reran the primary models. Then, we tested whether the transition to widowhood (the most common transition; *N* = 1941), was associated with the risk of dementia, compared to participants who were continuously married (reference). Two‐sided *P* < 0.05 was considered statistically significant.

## 3 RESULTS

### 3.1 Participants

At baseline assessment, 24,107 NACC participants (14,369 female, 9738 male) ranged in age from 50 to 104 years (mean = 71.79, standard deviation [SD] = 8.82). During the 18.44 years of follow‐up (median = 3.97 years; 122,478 person‐years), 4853 participants (20.1%) developed dementia (3729 [76.8%] AD, 341 LBD [7.0%], 246 VD [5.7%], and 184 FTLD [3.7%]). The numbers of AD, LBD, VD, and FTLD do not add up to the total number of all‐cause dementia because not all dementia cases had a cause‐specific diagnostic code. Of all incident dementia, 3389 (21.9%) cases were among the 15,409 married participants, 864 (21.9%) among 3939 widowed, 433 (12.8%) among 3360 divorced, and 167 (12.4%) among 1339 never married. At baseline, the 4853 individuals who were subsequently diagnosed with dementia were more likely to be female, older, married, living not alone, White, referred by professionals, motivated by clinical evaluation to visit ADRCs, depressed, diagnosed with hypertension, currently smoking, and with genetic vulnerability compared to individuals who were not diagnosed with dementia at follow‐up. Descriptive statistics of study variables are summarized in Table 1.

**TABLE 1:** Descriptive statistics of study variables for the full sample and by incident dementia.

		All‐cause dementia	
	Total	No	Yes
Variables, *N* (%)	24,107	19,254 (79.9)	4853 (20.1)
Sex			
Female	14,369 (59.6)	11,862 (61.6)	2507 (51.7)
Male	9738 (40.4)	7392(38.4)	2346 (48.3)
Age in years, mean (SD)	71.79 (8.82)	70.97 (8.72)	75.05 (8.43)
Education in years, mean (SD) (*n* = 24,020)	15.73 (3.10)	15.78 (3.06)	15.55 (3.24)
Race (*n* = 24,002)			
White	19,189 (79.9)	15,021 (78.4)	4168 (86.1)
Black/African American	3731 (15.5)	3254 (17.0)	477 (9.9)
Asian	659 (2.7)	534 (2.8)	125 (2.6)
Other	423 (1.9)	354 (1.8)	69 (1.4)
Ethnicity (Hispanic/Latino) (*n* = 24,023)	1767 (7.4)	1508 (7.9)	259 (5.3)
Living alone (*n* = 24,068)			
Alone	6843 (28.4)	5675 (29.5)	1168 (24.1)
not alone	17,225 (71.6)	13,544 (70.5)	3681 (75.9)
Diagnostic form			
UDS 1	961 (4.0)	625 (3.2)	336 (6.9)
UDS 2	7776 (32.3)	5469 (28.4)	2309 (47.6)
UDS 3	15,368 (63.7)	13,160 (68.4)	2208 (45.5)
Principal referral source (*n* = 23415)			
by professional	7547(32.2)	5434 (29.1)	2113 (44.8)
by non‐professionals	15,868 (67.8)	13,267 (70.9)	2601 (55.2)
Primary reason to visit ADRCs (*n* = 24,073)			
Participate to the study	19,356 (80.4)	15,804 (82.2)	3552 (73.3)
Clinical evaluation/both clinical evaluation and participate to the study	4717 (19.6)	3424 (17.8)	1293 (26.7)
Marital status			
Married	15,409 (63.9)	12,020 (62.4)	3389 (69.8)
Widowed	3939 (16.3)	3075 (16.0)	864 (17.8)
Divorced	3360 (13.9)	2927 (15.2)	433 (8.9)
Never‐married	1339 (5.8)	1232 (6.4)	167 (3.4)
Depression (active depression in the last 2 years) (*n* = 23,916)	5256 (22.0)	3850 (20.2)	1406 (29.2)
Diabetes (*n* = 24,013)	3137 (13.1)	2530 (13.2)	607 (12.5)
Obesity	5714 (23.7)	4899 (25.4)	815 (16.8)
Hypertension (*n* = 24,000)	12,014 (50.1)	9487 (49.5)	2527 (52.3)
Smoking			
Current (*n* = 23,486)	10,108 (43.0)	7431 (39.5)	2005 (42.7)
Never (*n* = 23,547)	14,223 (60.4)	11,403 (60.5)	2820 (59.8)
*APOE* ε4 present (*n* = 20,532)	7246 (35.3)	5179 (31.9)	2067 (48.2)

*Note*: In parentheses are SD or %.

Abbreviations: ADRC, Alzheimer's Disease Research Center; *APOE*, apolipoprotein E; SD, standard deviation; UDS, Uniform Data Set.

Of the four marital status groups, the widowed had an older age at baseline (Mean = 78.32, SD = 8.38) compared to the married (Mean = 70.73, SD = 8.34), divorced (Mean = 70.11, SD = 7.97), and never married (Mean = 69.14, SD = 8.97) groups. The widowed were also diagnosed at an older age (Mean = 85.61, SD = 7.87) compared to married (Mean = 77.42, SD = 8.72), divorced (Mean = 78.74, SD = 8.61), and never married (Mean = 79.87, SD = 10.11). Additionally, at the time of diagnosis, the widowed had more severe dementia (CDR Global score Mean = 0.86, SD = 0.50) compared to married (Mean = 0.79, SD = 0.44), divorced (Mean = 0.78, SD = 0.44), and never married (Mean = 0.78, SD = 0.44).

### 3.2 Cox proportional hazards regression results of marital status predicting all‐cause dementia

The Cox proportional hazards regression results are shown in Table 2. Surprisingly, all unmarried groups were at a lower risk of all‐cause dementia compared to the married group. The results indicated similar effects for widowed (hazard ratio [HR] = 0.73, 95% confidence interval [CI] = 0.67–0.79), divorced (HR = 0.66; 95% CI = 0.59–0.73), and never married (HR = 0.60, 95% CI = 0.52–0.71) groups. These associations were attenuated but remained significant in the fully adjusted model for divorced (HR = 0.83, 95% CI = 0.72–0.96) and never married (HR = 0.76, 95% CI = 0.62–0.93), but not for widowed (HR = 0.90, 95% CI = 0.79–1.03).

**TABLE 2:** Cox proportional hazards regression results of marital status predicting all‐cause dementia for unmarried groups.

		Reference (married)		
	All‐cause dementia	Widowed	Divorced	Never married
Model 1, HR (95% CI)	4853/24,107	0.73 (0.67–0.79)	0.66 (0.59–0.73)	0.60 (0.52–0.71)
Model 2, HR (95% CI)	3882/18,749	0.90 (0.79–1.03)	0.83 (0.72–0.96)	0.76 (0.62–0.93)
*Sensitivity analyses*				
Model 1.1, HR (95% CI)	3073/12,172	0.75 (0.67–0.84)	0.57 (0.50–0.65)	0.50 (0.40–0.62)
Model 1.2, HR (95% CI)	1780/11,935	0.81 (0.71–0.92)	0.82 (0.70–0.97)	0.83 (0.66–1.05)
*Participants—without cognitive impairment*				
Model 1, HR (95% CI)	1241/14,974	0.79 (0.68–0.92)	0.77 (0.63–0.94)	0.68 (0.50–0.91)
Model 2, HR (95% CI)	1055/11,855	0.69 (0.54–0.90)	0.75 (0.56–1.00)	0.64 (0.43–0.93)
*Progression from cognitive impairment* *to dementia*				
Model 1, HR (95% CI)	3612/9112	0.78 (0.70–0.86)	0.59 (0.52–0.67)	0.57 (0.47–0.68)
Model 2, HR (95% CI)	2827/6884	1.03 (0.87–1.21)	0.83 (0.69–0.98)	0.76 (0.60–0.96)
*Incident mild cognitive impairment*				
Model 1, HR (95% CI)	3133/14,517	0.97 (0.87–1.07)	1.10 (0.98–1.23)	1.09 (0.93–1.27)
Model 2, HR (95% CI)	2026/9977	0.95 (0.79–1.15)	1.17 (0.97–1.42)	1.02 (0.81–1.30)

*Note*: Model 1 include the covariates age and sex. Model 2 is Model 1 plus race, ethnicity, education, living alone, physical health (diabetes, hypertension, obesity), smoking behavior (current and former), depression, diagnostic form, participant's referral source, primary reason to visit ADRC, and *APOE* ε4.

Model 1.1 excludes participants with follow‐up time more than 4 years. Model 1.2 excludes participants with follow‐up time less than 4 years.

Abbreviations: ADRC, Alzheimer's Disease Research Center; *APOE*, apolipoprotein E; CI, confidence interval; HR, hazard ratio; MCI, mild cognitive impairment.

### 3.3 Cox proportional hazards regression results of marital status predicting AD, LBD, VD, FTLD

All unmarried groups were at lower risk of AD (widowed: HR = 0.69, 95% CI = 0.63–0.76; divorced: HR = 0.68, 95% CI = 0.60–0.76; never married: HR = 0.53, 95% CI = 0.51–0.73) and LBD (widowed: HR = 0.47, 95% CI = 0.30–0.74; divorced: HR = 0.21, 95% CI = 0.10–0.41; never married: HR = 0.26, 95% CI = 0.10–0.64) in the basic model. The associations remained significant in the fully adjusted model for AD (widowed: HR = 0.84, 95% CI = 0.72–0.98; divorced: HR = 0.84 = 95% CI = 0.71–0.99; never married: HR = 0.73, 95% CI = 0.58–0.91), but only for divorced for LBD (widowed: HR = 0.58, 95% CI = 0.27–1.24; divorced: HR = 0.33, 95% CI = 0.13–0.80; never married: HR = 0.46, 95% CI = 0.15–1.39). Divorced and never‐married groups were also at a lower risk for FTLD in the basic model (widowed: HR = 0.59, 95% CI = 0.32–1.08; divorced: HR = 0.54, 95% CI = 0.32–0.91; never married: HR = 0.29, 95% CI = 0.11–0.80), but not in the fully adjusted model (widowed: HR = 1.08, 95% CI = 0.46–2.54; divorced: HR = 0.75, 95% CI = 0.33–1.72; never married: HR = 0.50, 95% CI = 0.14–1.76). There was no significant association for VD in the basic (widowed: HR = 0.98, 95% CI = 0.70–1.36; divorced: HR = 0.65, 95% CI = 0.39–1.09; never married: HR = 0.67; 95% CI = 0.32–1.38) and fully adjusted model (widowed: HR = 0.93, 95% CI = 0.55–1.59; divorced: HR = 0.53, 95% CI = 0.25–1.10; never married: HR = 0.75, 95% CI = 0.32–1.78; Table 3).

**TABLE 3:** Cox proportional hazards regression results of marital status predicting AD, LBD, VD, FTLD.

	Reference (married)			
	AD	LBD	VD	FTLD
Model 1, # dementia	3729/24,101	341/24,035	246/24,080	184/24,107
Model 1, HR (95% CI)				
Widowed	0.69 (0.63–0.76)	0.47 (0.30–0.74)	0.98 (0.70–1.36)	0.59 (0.32–1.08)
Divorced	0.68 (0.60–0.76)	0.21 (0.10–0.41)	0.65 (0.39–1.09)	0.54 (0.32–0.91)
Never married	0.53 (0.51–0.73)	0.26 (0.10–0.64)	0.67 (0.32–1.38)	0.29 (0.11–0.80)
Model 2, # dementia	2998/18,746	251/18,705	193/18,733	155/18,749
Model 2, HR (95% CI)				
Widowed	0.84 (0.72–0.98)	0.58 (0.27–1.24)	0.93 (0.55–1.59)	1.08 (0.46–2.54)
Divorced	0.84 (0.71–0.99)	0.33 (0.13–0.80)	0.53 (0.25–1.10)	0.75 (0.33–1.72)
Never married	0.73 (0.58–0.91)	0.46 (0.15–1.39)	0.75 (0.32–1.78)	0.50 (0.14–1.76)

*Note*: Model 1 include the covariates age and sex, Model 2 is Model 1 plus race, ethnicity, education, living alone, physical health (diabetes, hypertension, obesity), smoking behavior (current and former), depression, diagnostic form, participant's referral source, primary reason to visit ADRC, and *APOE* ε4.

Abbreviations: AD, Alzheimer's disease; ADRC, Alzheimer's Disease Research Center; *APOE*, apolipoprotein E; CI, confidence interval; FTLD, frontotemporal lobar degeneration; HR, hazard ratio; LBD, Lew body disease; VD, vascular dementia.

### 3.4 Moderation

Interaction terms were tested to evaluate whether sex, age, race, education, depression, diagnostic form, referral source, the primary reason to visit ADRC, and *APOE* ε4 moderated the associations between marital status (unmarried vs. married) and risk of dementia. There was some evidence of moderation by age (interaction HR = 1.02, 95% CI = 1.01–1.02), sex (interaction HR = 0.79, 95% CI = 0.68–0.91), and referral source (interaction HR = 0.77, 95% CI = 0.67–0.87), the other interactions were not significant (Table S1 in supporting information). Stratified analyses showed small differences between groups, suggesting that associations were apparent across groups with just some differences in strength across age (age ≤ 72 HR = 0.58, 95% CI = 0.52–0.64; age > 72 HR = 0.71, 95% CI = 0.65–0.77), sex (female HR = 0.69, 95% CI = 0.63–0.75; male HR = 0.59, 95% CI = 0.52–0.67), and referral source (referred by professional HR = 0.64, 95% CI = 0.57–0.71; referred by non‐professional HR = 0.75, 95% CI = 0.68–0.82).

### 3.5 Sensitivity analysis

Accounting for age and sex, the associations between the unmarried subgroups and risk of all‐cause dementia were similar when follow‐up was < 4 years (widowed: HR = 0.75, 95% CI = 0.67–0.84; divorced: HR = 0.57, 95% CI = 0.50–0.65; never married: HR = 0.50, 95% CI = 0.40–0.62). When follow‐up was > 4 years, widowed and divorced were associated with a lower risk of dementia (widowed: HR = 0.81, 95% CI = 0.71–0.92; divorced: HR = 0.82, 95% CI = 0.70–0.97), whereas never married was not associated with dementia risk (HR = 0.83, 95% CI = 0.66–1.05). The results from the competing risk analyses that accounted for 2795 deaths were also similar (widowed: HR = 0.71, 95% CI = 0.65–0.77; divorced: HR = 0.64, 95% CI = 0.58–0.70; never married: HR = 0.59, 95% CI = 0.51–0.69). In analyses that excluded participants with cognitive impairment–MCI (*n* = 1449) or MCI (*n* = 7663) at baseline, unmarried participants were still at lower risk of dementia (widowed: HR = 0.79, 95% CI = 0.68–0.92; divorced: HR = 0.77, 95% CI = 0.63–0.94; never married: HR = 0.68, 95% CI = 0.50–0.91) relative to married participants in the basic model. These associations were attenuated but remained significant in the fully adjusted model for widowed (HR = 0.69, 95% CI = 0.54–0.90) and never married (HR = 0.64, 95% CI = 0.43–0.93), but not for divorced (HR = 0.75, 95% CI = 0.56–1.00; Table 2).

In the sensitivity analysis that included only participants with any MCI at baseline, unmarried participants were still at a lower risk of dementia (widowed: HR = 0.78; 95% CI = 0.70–0.86; divorced: HR = 0.59; 95% CI = 0.52–0.67; never married: HR = 0.57; 95% CI = 0.47–0.68) in the basic model. The association was attenuated but remained significant in the fully adjusted model for divorced (HR = 0.83, 95% CI = 0.69–0.98) and never married (HR = 0.76, 95% CI = 0.60–0.96), but not for widowed (HR = 1.03; 95% CI = 0.87–1.21) participants.

We next examined marital status and risk of incident MCI. For this analysis, we excluded both participants with any cognitive impairment at baseline and those who progressed directly to dementia (*n* = 470). All unmarried groups were unrelated to MCI risk (widowed: HR = 0.97, 95% CI = 0.87–1.07; divorced: HR = 1.10, 95% CI = 0.98–1.23; never married: HR = 1.09, 95% CI = 0.93–1.27) in Model 1. The associations were similar in the fully adjusted model (widowed: HR = 0.95, 95% CI = 0.79–1.15; divorced: HR = 1.17, 95% CI = 0.97–1.42; never married: HR = 1.02, 95% CI = 0.81– 1.30). Finally, in a supplemental analysis, we excluded participants who experienced marital transitions over the study follow‐up (*N* = 2916). After accounting for age and sex, the results were unchanged (*N* = 21,084; incident dementia *N* = 4202; widowed: HR = 0.65, 95% CI = 0.59–0.71; divorced: HR = 0.58, 95% CI = 0.52–0.65; never married: HR = 0.51, 95% CI = 0.43–0.61). Then, we examined the risk of dementia among participants who transitioned to widowed across the waves (*N* = 1941) while keeping all other coding consistent with the primary analysis. Accounting for age and sex, participants who experienced transitions in widowhood had a lower risk of dementia compared to those consistently married (HR = 0.52, 95% CI = 0.47–0.57).

## 4 DISCUSSION

The present study examined the association between marital status and the risk of dementia in a US sample of older adults followed for up to 18 years. Accounting for age and sex, we found that widowed, divorced, and never‐married individuals had ≈ 50% or lower dementia risk relative to their married counterparts. The associations held for divorced and never‐married older adults after controlling for age, sex, race, ethnicity, education, living alone, depression, smoking behavior, diabetes, hypertension, obesity, diagnostic form, participant's referral source, reason to visit ADRC, and *APOE* ε4. All unmarried groups were also at a lower risk of AD and LBD, with a pattern of mixed findings for FTLD, and no associations with risk of VD or MCI. Follow‐up analyses found evidence that all unmarried individuals were still at a lower risk of all‐cause dementia relative to married participants after excluding MCI at baseline. And among those with MCI at baseline, the unmarried group was at lower risk of progression from MCI to dementia. There was some evidence of moderation by age, sex, and referral source. However, stratified analyses showed small differences between groups, and most interactions were not significant, suggesting that the role of marital status in dementia tends to be similar across individuals at different levels of dementia risk due to their education, depression, and genetic vulnerability. Accounting for competing risk of death had no impact on the results, which supports the robustness of the findings.

Our findings that all unmarried groups (widowed, divorced, never married) were associated with a lower dementia risk relative to married participants is contrary to most previous longitudinal studies, which have reported that married individuals have a lower risk of cognitive impairment and dementia than unmarried groups.^5^, ^8^, ^39^ However, another population‐based study from the United States did not report the advantages of being married relative to never married in terms of cognitive impairment or dementia,^10^ and a recent US study suggested improvement in cognition and less cognitive decline after divorce.^16^ There is some evidence indicating an increase in some domains of well‐being, such as happiness and life satisfaction, after divorce^12^, ^13^ and social participation after partner bereavement.^40^ Never‐married individuals are also more likely to socialize with friends and neighbors^41^ and are more likely to engage in healthier behaviors than their married counterparts.^42^ Married individuals tend to have less social integration^41^ and are engaged in less frequent and lower‐quality interactions in their networks compared to their unmarried counterparts.^43^ These positive aspects of well‐being and social ties may potentially serve as protective factors against dementia over time.^14^, ^15^, ^44^ There is substantial evidence that the health benefits of marriage appear to be only in high‐quality marriages.^45^ In contrast, individuals who are unhappy in their marriage, an indicator of marital quality, are more likely to have equal or worse health and mortality risk compared to those who are widowed, divorced, or never‐married counterparts.^46^ Thus, marital quality may play a key role in the association.^47^ While these psychosocial factors could be plausible mechanisms, our findings suggest that vascular factors are less likely to explain the associations. Indeed, we found no significant associations with VD, and accounting for vascular risk factors had little impact on the overall associations. Future research is needed to identify the mechanisms linking marital status and incident dementia.

The finding that unmarried individuals in NACC were less likely to be diagnosed with dementia could be due to an ascertainment bias, with married individuals more likely to have partners who notice and report cognitive failures.^25^ A population‐based cohort study found that bereaved older individuals were less likely to have a dementia diagnosis in their health records over 20 years of follow‐up, suggesting that widowed individuals were underdiagnosed in routine clinical care relative to their partnered counterparts.^30^ Individuals may be unaware of their symptoms, particularly in the early stages of dementia.^25^ The subtle prodromal changes associated with cognitive impairment and dementia, such as memory, personality, and behavior changes,^48^, ^49^, ^50^ are frequently first reported by partners/spouses. Thus, it is possible that married individuals are more likely to seek a dementia evaluation and to be diagnosed at an earlier stage compared to those who are unmarried. The possibility that unmarried individuals are more likely to be delayed/missed diagnosed with dementia due to a lack of referrals from spouses raises concerns about the possibility of later‐stage dementia diagnosis or undiagnosed dementia among unmarried individuals. Interestingly, however, the moderation analyses indicated that the associations were similar by age or referral source, with slightly weaker associations among individuals who were older and referred to the clinic by non‐professionals, such as self, family, or friends. Furthermore, while the widowed were diagnosed at an older age and with slightly more severe symptoms compared to the other groups, the never‐married and divorced did not differ from the married on the age or severity of impairment at the time of diagnosis. Overall, it is unclear why such bias (delayed diagnosis in the unmarried) would affect the findings from the NACC and no other studies. The NACC annual assessments with standardized protocols make such bias less likely compared to studies that rely on less frequent assessments or health records.

The moderation analysis indicated that the association between marital status and incident dementia did not vary across race, education, depression, the presence of the *APOE* ε4 allele, and the primary reason for visiting the ADRCs. This finding is consistent with a recent study that found that race, education, and *APOE* ε4 did not significantly moderate the association between divorce and widowhood and the risk of dementia.^23^ There was also no significant interaction with the diagnostic form used, with consistent findings before and after the introduction of the CDR in 2015, suggesting that the associations remained similar despite changes in diagnostic methods and advancements in diagnostic capabilities over the past decade. Age, sex, and referral source did moderate the association between marital status and lower risk of dementia. For example, being unmarried was slightly more protective for males compared to females, or in other words, being married was associated with a slightly higher risk of dementia among males more than females. However, stratified analyses showed small differences between groups, suggesting that the protective role of being unmarried was similar across older and younger adults, males and females, and those referred by professionals or non‐professionals.

In the sensitivity analysis, we found that marital status is unrelated to incident MCI, while it is associated with a lower risk of dementia as well as progression from MCI to dementia. Despite the specific reasons for this variation across cognitive outcomes remaining unclear, this finding aligns with a previous study using the NACC database suggesting that social relationships, including marital status, may not be a strong independent clinical predictor of MCI.^9^ We also found that participants who experienced widowhood during the study follow‐up were at a lower risk of dementia than those who remained continuously married. Although the transition to widowhood is a challenging life event, it may also reduce chronic stress for some older adults, such as those who face marital strain or the burden of long‐term caregiving. Widowhood can also lead to an increase in close network size in the post‐widowhood years,^51^ which may potentially protect against dementia risk.

The present study has several strengths, including rigorous cognitive assessment and clinical diagnoses of dementia in specialized centers across the United States. The large sample, older age, assessment of dementia risk factors, the long follow‐up, and the analyses of cause‐specific dementia are other considerable strengths. The study has also some limitations. First, the current study includes referral‐based or voluntary NACC participants who do not represent the US population, particularly in much older ages, more educated, and worse subjective cognition.^52^ Additionally, the majority of the sample was White and married, with a relatively small proportion of Black and unmarried individuals. As such, an important future direction is to examine the specific pathways that lead to reduced dementia risk among unmarried older adults over time in a more representative sample. For example, future work would benefit from examining other socioeconomic status factors, apart from educational attainment, because of the potential indirect association between marital status and dementia through economic resources.^5^ We would note that the NACC will provide a module with the UDSv4, focusing on various social determinants of health, including financial stress, social connections, and health‐care experiences, which will provide an opportunity to examine such possible pathways between marital status and the risk of dementia. Furthermore, examining the association through duration of post‐marital life, including transitions to divorce, with the role of relationship factors (e.g., marital quality, relationship duration) may provide a more nuanced understanding than a simple binary effect. In conclusion, using the large NACC cohort, this study found married older adults have a higher risk of dementia compared to never‐married, divorced, and widowed adults. The findings could be due to delayed diagnoses among unmarried individuals or present a challenge to assumptions that being married provides protection against dementia.

## CONFLICT OF INTEREST STATEMENT

The authors declare no competing interests. Author disclosures are available in the supporting information.

## Supporting information



Supporting Information

Supporting Information

## Data Availability

The NACC data are available to researchers who apply with a NACC data request (https://naccdata.org/requesting‐data/data‐request‐process). Key elements of the application include a research plan, the goal of the analysis using NACC data, and a listing of requested variables.
