# Supplementary material for: Marital status and risk of dementia over 18 years: Surprising findings from the National Alzheimer's Coordinating Center
Source: Alzheimers Dement. 2025 Mar 20;21(3):e70072. doi: 10.1002/alz.70072 (PMC11923573; doi:10.1002/alz.70072)
Supplement: Supplementary file 2 — Supporting Information [file ALZ-21-e70072-s001.docx]

**TABLE S1.**

*Cox regression results of interaction between age, sex, race, education, depression, diagnostic form, referral source, primary reason to visit ADRC, and APOE* ϵ4 *in marital status indicators.*

|  | Cases (n) / total (No.) | HR (95% CI) |
| --- | --- | --- |
|  | Reference (married) | |
| Interaction with age | 4853/24107 | 1.02 (1.01-1.02) |
| *≤ 72 years* | 1791/12992 | 0.58 (0.52-0.64) |
| > *72 years* | 3062/11109 | 0.71 (0.65-0.77) |
| Interaction with sex | 4853/24107 | 0.79 (0.68-0.91) |
| *Female* | 2507/14367 | 0.69 (0.63-0.75) |
| *Male* | 2346/9738 | 0.59 (0.52-0.67) |
| Interaction with race (White) | 4839/24002 | 0.89 (0.55-1.44) |
| Interaction with race (Black) | 4839/24002 | 1.17 (0.70-1.94) |
| Interaction with race (Asian) | 4839/24002 | 1.20 (0.65-2.21) |
| Interaction with education | 4835/24020 | 0.99 (0.97-1.01) |
| Interaction with depression | 4813/23916 | 0.95 (0.83-1.09) |
| Interaction with diagnostic form | 4853/24107 | 1.02 (0.90-1.15) |
| Interaction with referral source | 4714/23415 | 0.77 (0.67-0.87) |
| *By professions* | 2113/7547 | 0.64 (0.57-0.71) |
| *By non-professionals* | 2601/15866 | 0.75 (0.68-0.82) |
| Interaction with primary visiting reason | 4845/24073 | 0.88 (0.76-1.01) |
| *APOE* ϵ4 | 4286/20532 | 0.91 (0.79-1.03) |

*Note.* Age and sex are covariates in all models. Abbreviations: HR=Hazard Ratio; CI=Confidence Interval.
